# Preschoolers Build Fundamental Motor Skills Critical to an Active Lifestyle: The *All 4 Kids^©^* Intervention Study

**DOI:** 10.3390/ijerph17093098

**Published:** 2020-04-29

**Authors:** Anne R. Lindsay, Angela Starrett, Ali Brian, Teresa A. Byington, Jennifer Lucas, Madeleine Sigman-Grant

**Affiliations:** 1 Extension, College of Agriculture, Biotechnology & Natural Resources (CABNR), University of Nevada, Reno, NV 89123, USA; byingtont@unr.edu (T.A.B.); sigmangrantm@unr.edu (M.S.-G.); 2 Department of Educational Studies, College of Education, University of South Carolina, Columbia, SC 29208, USA; starrett@mailbox.sc.edu; 3 Department of Physical Education, College of Education, University of South Carolina, Columbia, SC 29208, USA; abrian@sc.edu; 4 Department of Family Medicine, Oregon Health & Science University, Portland, OR 97239, USA; lucasje@ohsu.edu

**Keywords:** early childhood, movement, physical development, physical activity, motor development, gross motor, dance, perceptual motor

## Abstract

This pragmatic, real world study examined the effects of the *All 4 Kids*^©^ intervention on preschoolers’ mastery of movement skills and determined whether the instruction had greater impact than natural development. Methods included a quasi-experimental intervention-comparison subsample of 379 children (COMPARISON) and a pretest-posttest design with convenience scale-up sampling of 2817 preschoolers (SCALE-UP). Children receiving education and dance instruction 3 times/week for 8 weeks were assessed using the Preschool Movement Assessment to evaluate skills pre and post intervention. Using repeated measures ANOVA, McNemar and Wilcoxon signed ranks tests, preschooler’s participation in the intervention resulted in greater improvement in 12 movement skills (*F* = 83.451, *df* = 1, *p* < 0.001, $\eta_{p}^{2}$ = 0.555), balance (*p* = 0.028), hopping (*t* = −3.545, *df* = 112, *p* = 0.001) and crossing the midline (*p* < 0.001) than natural development (COMPARISON). In the SCALE-UP study, children significantly improved in all measures based on post-intervention scores. Significant differences were observed between Hispanic and non-Hispanic children for the 12-skills (*b* = −0.758, *se* = 0.161, *p* < 0.001) using hierarchical linear models; boys’ and girls’ scores were not differentially impacted by the intervention. Therefore, implementation of interventions focused on fundamental movement skill development have the potential to remediate secular motor skill decline in young children.

## 1. Introduction

While numerous factors contribute to obesity in young children, low levels of physical activity play a particularly significant role [1]. Contrary to popular belief that children in childcare programs are generally active, there is evidence to suggest that activity levels in these programs are low [2,3,4,5]. Typically, four- and five-year-old children tend to be less active than three-year-olds [6] with physical activity (specifically step counts) declining steadily after age six [7]. Children who are proficient in fundamental motor skills are not only more likely to be physically active than children that are less proficient, [8] but also are more likely to engage in vigorous physical activity as adolescents [9].

In addition to promoting physical activity, skill mastery is critical to the overall development of the child [8,10,11,12,13]. Delivering physically active *lessons* to children ages 5–13 rather than simply increasing activity time resulted in greater improvements in academic achievement [14]. Moreover, while both cardio-activity (e.g., running with increased heart rate) and motor/coordinative exercises (e.g., balancing on an unstable surface) are important for potentially improving children’s cognition, motor-demanding exercise bouts in 9–10-year-olds can lead to better performance in concentration and attention tasks than cardio alone [11]. Therefore, interventions that integrate intentional movements accompanied by explicit instructions from trained adults should reflect both cognitive and physical developmental milestones associated with young children (3–6 years) [12,14,15,16,17].

Mastering basic movement skills (e.g., locomotion [run, jump, hop], stability [twist, bend] and object manipulation [throw, catch, kick]) is purported to promote “physical literacy,” broadly defined as building a child’s capacity to engage in a physically active lifestyle. The International Physical Literacy Association, [18] defines physical literacy as the “motivation, confidence, physical competence, knowledge, and understanding to value and take responsibility for engagement in physical activities for life.” It is critical to be motivated to perform these skills and do so with confidence to establish a physically active lifestyle [8,9,19,20]. Children must develop competency in performing basic fundamental motor skills while simultaneously building confidence in these abilities in order to have a positive effect on increasing physical activity, especially during their adolescent years [9,20,21,22]. Such mastery of fundamental motor skills should begin in early childhood [8,10,11].

Although the mastery of fundamental motor skills should begin in early childhood, many children in the United States fail to do so. In fact, today’s preschoolers may be experiencing a secular decline with their fundamental motor skills when compared to normative references from 20–30 years ago [23]. As such, as many as 78% within any given sample of US preschoolers show developmental delays with their fundamental motor skills [15,16,17,23,24]. Unfortunately, young girls tend to be at greater risk for developmental delays than young boys, warranting a special attention.

To remediate this secular motor skill developmental decline and delay in US preschoolers, many different intervention programs exist. Within the motor skill intervention literature, there is much variance regarding the rigor, effectiveness and generalizability of the programs and their outcomes. For the most part, motor skill intervention programs are relatively effective at improving motor skills [25,26,27]. However, the results of many of these studies should be interpreted with caution as many are efficacy trials, conducted under ideal circumstances, which may not be generalizable in other settings, i.e., the implementer of the intervention matters. Within most efficacy motor skill trials, an expert in motor development, with a doctorate, will lead the intervention. Unfortunately, less than 1% of early childhood centers actually employ staff with degrees in physical education and/or expertise in motor development [28,29]. Thus, translating many of the efficacy trials into scalable practice is relatively challenging.

Not only is it difficult to translate most motor skill intervention research into practice, but many findings are not generalizable due to small, homogenous sample sizes, [25,26,27] thus limiting statistical analyses. Typically, factors such as school location, teacher effects and the extent to which the intervention meets state-level standards, cannot be determined without a sample size big enough to power nested models. Finally, the differential effects of many published motor skill interventions for sex is also unclear [25,26,27].

Developers of the *All 4 Kids: Healthy, Happy, Active, Fit^©^* Program (herein, *All 4 Kids*^©^) determined that the use of dance as an intervention implemented by teachers and reinforced by parents, would address the ecological validity issues present within the motor skill intervention literature. To achieve these fundamental and coordinative movement goals for young children [30], *All 4 Kids^©^* provides opportunities for adult-led, structured physical activity to improve fundamental movement skills that form the building blocks for motor skill development. Moreover, partnering with teaching staff and parents and families increases the likelihood of enhanced participation and maximizes potential sustainability [25]. In addition, this program addresses extant gaps in the following ways:

- The intervention is intended for preschool-age children, specifically, ages four and five with a primary focus on children who cannot perform basic motor skill developmental standards (early mastery)
- The intervention utilizes dance as the primary activity (crossing genre, generations, sex and ethnicity)
- The dances incorporate Nevada’s Pre-Kindergarten Physical Development Standards [31] (e.g., cross the midline, step backwards, spin on one foot, etc.)
- The dances allow for learning inclusion regardless of skill level (differentiated learning)

Although *All 4 Kids^©^* is highly rigorous, the extent to which *All 4 Kids^©^* supports standards-based learning for boys and girls must be determined. Therefore, the purposes of this study were: (1) to conduct an ecologically valid trial to distinguish between intervention and comparison groups and determine the impact of directed movement vs. natural development of motor skills in preschoolers (COMPARISON); and (2) to examine the effects of the *All 4 Kids*^©^ intervention on the mastery of movement skills in a large, scaled-up sample of preschoolers located throughout Southern Nevada (Scale-up). It was hypothesized that the intervention children in the COMPARISON study would show greater improvements in movement skills than the effects of development alone (comparison group) and that preschoolers receiving the *All 4 Kids*^©^ intervention in the Scale-Up study would significantly improve their movement skills.

## 2. Materials and Methods

### 2.1. Intervention Program

The original impetus for *All 4 Kids*^©^ in 2007 was to address the rising rates of early childhood overweight and obesity. Three academicians/practitioners combined their expertise from the fields of child nutrition, public health, child development and exercise physiology to systematically construct a nine-week program delivered to preschool-aged children, their early childhood education (ECE) providers and their parents. The primary objectives of the *All 4 Kids*^©^ program addressed physical activity, practice and mastery of fundamental movement skills, promotion of healthy snacks and acceptance of body shape and size. The program and the nutrition education component were previously described [30,32,33].

The *All 4 Kids*^©^ physical activity and skill mastery components were addressed through dance choreography. Original songs were written and recorded that include specifically designed dance patterns that teach children a variety of perceptual motor development and fundamental movement skills and incorporate many of Nevada’s Pre-Kindergarten physical development standards [31]. One focused dance was taught in each of the 3 units including country line dance (Unit 1), Hip-Hop (Unit 2) and Salsa (Unit 3). Each of the 21 lessons included a focused physical development movement (e.g., sway, hop, step back, etc.) that was also part of the dance choreography. Music and dances are multi-genre, multi-cultural and incorporate healthy messaging through lyrics. Visual instructions are provided to make it engaging for both the preschoolers as well as the adults in their lives.

Choreographed movements selected were reflective of a self-driven, motor learning practice based on the Self-Determination Theory [34]. Movements included fundamental movement skills (jump, hop, spin, cross feet, etc.); proprioception and perceptual motor skills (e.g., shrugging the shoulders, moving the hips, tapping the heel); directional movements (forward, backward, sideways); speed (move fast, move slow) and spatial awareness (whole body movement, crossing the midline, etc.) [10,35,36]. Additionally, dances incorporated other basic pre-Kindergarten curriculum standards including math (such as counting), music and creative arts.

The original program was nine weeks in length and consisted of 24, 30–35 min-lessons taught three times per week in preschool classrooms by University instructors or preschool teachers trained in the curriculum. In 2013, the program was reduced from 9 weeks/24 lessons to 8 weeks/21 lessons (lessons in Unit 3 that focus on acceptance of body shape and size were reduced from eight to five).

### 2.2. Research Design

This study featured a quasi-experimental pretest-posttest design with convenience sampling across two phases between 2010 and 2016. The variable tested was the impact of the skill mastery component of the *All 4 Kids*^©^ program on children attending preschool. This study was guided by the social-ecological model [37] that provides a framework for understanding how the individual interacts with the context (e.g., microsystem—e.g., parents, teachers, schools, community). According to this model, skill development results from the complex interrelation between multiple factors within individuals and their environment. Lessons were designed to include differentiated learning using a variety of teaching techniques and lesson adaptations across multiple skill levels in the classroom (teacher), the home (family) or other learning environments (community).

### 2.3. Setting and Participants

#### 2.3.1. Recruitment

Since both studies were pragmatic and not experimental by design, recruitment was with the centers and not the individual participants. All preschoolers in the centers for both COMPARISON and SCALE-UP studies were eligible and recruited through parent orientations. All children were assessed both pre- and post- intervention unless the parent or the child did not want to be assessed (in which case, the child still received the program) or the child was absent during the assessment.

#### 2.3.2. Comparison Study (COMPARISON)

A subsample of preschools in Clark County, Nevada was chosen from a convenience sample of 12 Head Start childcare centers in 2010. Head Start centers were chosen for the COMPARISON study because they were similar (less variability) across centers. Limited variation between schools is attributed to uniformity in organizational structure and administrative oversight and tight quality control. All 12 centers were stratified based on primary parental language (English = 6; Spanish = 6) and ordered by size within language group. Centers then were matched based on size. There were 379 children, aged 4 (59% in the intervention and comparison groups) or 5 (41% in the intervention and comparison groups). The COMPARISON subsample excluded 3 and 6-year-olds as the intervention was designed for 4 and 5- year-olds.

Centers assigned to the intervention group received the *All 4 Kids*^©^ program (not previously exposed), which was designed to incorporate state pre-K physical development standards into the physical activities; whereas preschools assigned to the comparison group received their usual physical activity programming based on national or local childcare recommendations.

#### 2.3.3. Scale-Up Study (SCALE-UP)

In the second phase of the pragmatic study, 28 preschools were selected from a convenience sample and rotated every session so as not to duplicate research or contaminate participant data (included in the sample were the original 12 Head Start Centers, now with different children). The SCALE-UP population was comprised of 2817 children who all completed the *All 4 Kids^©^* intervention program during spring 2011 through spring 2016 in Nevada. The SCALE-UP subsample included all children ages three to six as is ordinarily found in a preschool classroom.

### 2.4. Instrumentation

A Preschool Movement Assessment (PMA), designed by the authors to gauge a preschooler’s coordination and movement ability, [38] was administered to all children in a pre- and post-assessment manner. Commonly used tools (such as the Test of Gross Motor Development or TGMD [39]) are designed for studies or clinical evaluation and require skilled technicians, space and time. Since this was a pragmatic study conducted in preschools as part of an obesity prevention initiative, SCALE-UP assessments were conducted in the classrooms by trained preschool teachers and staff hired to conduct the program. For the COMPARISON study, assessors were blinded to the treatment. External student hires from a nearby physical therapy school were recruited to conduct pre- and post- intervention assessments and rigorously trained [38]. Assessors were not involved in either instruction of the program or in the study itself and did not know which schools were assigned to either cohort.

The PMA required each preschooler to perform a variety of movements divided into 4 tasks, including a baseline of 12 basic skills (“12-skills”) such as spinning on one foot, jumping, hopping on one foot and stepping backwards. This was followed by 3 additional tasks: timed “balance,” “boxing” (to assess mid-line crossing) and number of “hops” completed (to assess endurance and coordination). The 12-skills and boxing task were clearly delineated in the tool’s scoring instructions and coded as “competent,” (a score of 3), “not-competent” (a score of 2) or “unable to perform” (a score of 1). “Unable to perform” simply means the child performed the skill incorrectly or did not know how to perform the skill. (Note: factors such as language or unable to understand what was being asked were both guided by the instrument protocol.) “Not competent” means the child could perform the skill but lacked competency or completeness based on the protocol scoring interpretation (e.g., the child was able to spin on 1 foot but only completed a 25% rotation).

Typically scores of 1–3 are used to evaluate changes in the child’s performance. However, for this analysis, scores (1–3) were dichotomized (0/1) as “completed the skill” (score of 1) or “did not complete the skill” (score of 0) by consolidating the “unable to perform” and “not competent” groups. By summing the 12-skills, a composite score ranging from 1–12 was created. This scoring, based on the state PreK Standards, demonstrates whether the child can perform the skill “yes” or “no” prior to kindergarten. Other tasks included balancing on one foot (continuously measured time, using only the right foot for a maximum of 30 s) based on the 5-s state pre-K standard; and number of hops on one foot in 15 s (continuous). For additional information about the PMA and its administration, please refer to the original manuscript [38].

### 2.5. Procedures

One trained instructor went into each preschool classroom three times weekly to deliver lessons and engage the children and their ECE teachers. While the original program was intended for two trained instructors, funding limitations only allowed for one instructor and assistance from teachers or teaching assistants. A CD was provided to allow teachers and children to practice the dances in the classroom between formal lessons and a music video (DVD) was provided to encourage children and their families to practice the dances at home. Prior to being allowed to interact with preschoolers, *All 4 Kids*^©^ teachers needed to demonstrate their own mastery of the movement skills (e.g., the dance steps) to ensure consistency across classrooms. Moreover, ECE teachers needed to learn these skills so that they could assist their students during regular classroom time. To achieve fidelity, teachers received in-person training, supplemented with on-line lessons and training videos that clearly explained and demonstrated the physical activity movement within the dance choreography (https://extension.unr.edu/healthykids/default.aspx).

Information sheets were provided to primary caregivers that discussed the *All 4 Kids*^©^ program and the purpose of the research. An invitation to participate was provided to all preschooler families. Consent was obtained from caregivers who allowed their children to be involved in the study. Those children who did not want to be assessed (or were not permitted by their parents) were excluded from the research, but not from participation in the intervention. The study was approved by the Institutional Review Board of the University of Nevada, Reno (#S12–081). Pre-data were collected 1–2 weeks prior to the beginning of the program and post-data were collected 1–2 weeks after program conclusion during spring, summer and fall sessions over the study period.

### 2.6. Statistical Analysis

Population demographic characteristics included: age in years, sex (male/female) and race/ethnicity (White/Caucasian, Black/African American, Hispanic/Latino and other). Race and ethnicity were recoded dichotomously to Hispanic/Latino and non-Hispanic for multiple models to ensure adequate power. Other characteristics such as BMI percentile were included. Although less than 1% of either pre- or post-intervention data were missing, full-information maximum likelihood estimation in Mplus [32,34,35,36] was applied.

For the COMPARISON study, repeated measures analysis of variance (ANOVA) was conducted to assess the difference between pre- and post- intervention 12-skill composite scores between groups. Since improvements for children who are unable to perform skills at pre-test is fundamental to the design of the program, McNemar tests were conducted to determine how many children unable to perform at pre-test succeeded at post-test in each of the 12 PMA skills separately. Balance was analyzed pre- and post- intervention using Wilcoxon signed ranks test for the comparison and intervention groups. A Mann Whitney U test was conducted to assess post-intervention balance between the intervention and comparison groups. A McNemar test was conducted to determine the number of children who could not balance for 5 s and whether or not they reached the Nevada standard. Crossing the midline was assessed pre- and post- intervention using McNemar tests for each group and with a Chi-squared test to compare the groups post-intervention. Hops were measured pre- and post- intervention using paired t-tests. An independent sample t-test was conducted to assess the intervention and comparison groups post-intervention.

For the SCALE-UP study, children were nested within numerous childcare centers (*n* = 28), so hierarchical linear modeling was used to separately examine the variation in score differences for the pre-intervention and post-intervention tasks of the 12-skills, boxing, hops and balance. Intraclass correlation coefficients (ICC) for the PMA skills, boxing and number of hops show significant nesting of children in childcare centers. Due to a non-normal distribution, the MLR estimator was used in Mplus with TYPE = COMPLEX. MLR produces maximum likelihood parameter estimates with standard errors and a chi-squared test statistic that are robust to non-normality of observations. For each model, ICCs that represent the proportion of variance in the task scores explained by the grouping structure of the hierarchical model were reported. Explanatory variables included sex, BMI z-score, ethnicity (dichotomized as 0-non-Hispanic, 1-Hispanic) and age (years). To complement the hierarchical linear model investigations, Wald tests were utilized to investigate the significant impact of the intervention on each of the task, as well as to determine if the impact was moderated by gender. All tests were run in Mplus version 8.1 [40] (Muthén & Muthén, Los Angeles, CA, USA). Using G*Power 3.1, a post hoc power analysis shows this study was fully powered to detect effect sizes as small as 0.10 given a Type I error rate of 5%.

## 3. Results

### 3.1. COMPARISON Results

Table 1 shows descriptive results of the sample from the COMPARISON study. Among the intervention group, 55.3% were female and 69.9% were Hispanic/Latino. In the comparison group, 59.8% were female and 72.8% were Hispanic/Latino. Of note, the percent of children in the intervention group that were in the obese or overweight percentile category (>85th percentile) was 41.5%. Among the comparison group, 47.6% of children had a BMI percentile in the obese or overweight category.

PMA 12-Skills. The repeated measures ANOVA showed that after controlling for age, sex, race/ethnicity and BMI z-score, participation in the intervention was statistically significant between the intervention and comparison groups (F = 83.451, df = 1, *p* < 0.001, $\eta_{p}^{2}$ = 0.555) with regards to the 12-skill composite scores. The interaction of time and treatment group confirmed that increases in skills was not due to maturation alone (F = 20.662, *df* = 1, *p* < 0.001, $\eta_{p}^{2}$ = 0.236). Overall, children in the intervention group had a PMA composite score mean change of 4.029 compared to 1.029 in the comparison group. For more detailed analyses, we considered specifically examining the impact on those children who were not competent or able to reach the pre-Kindergarten standard at pre-test. As such, children in the intervention group had a PMA composite score mean change of 7.225 and significant change in ten skills, and those in the comparison group had a mean change of 1.892 and significant change in only six skills pre and post-test (Table 2).

Balance (timed). With regards to balance, we first considered all of the children in the COMPARISON study. The children in the comparison group did not have a statistically significant change in balance, while the children in the intervention group did increase significantly from a mean of 5.32 s pre-intervention to 6.42 s post-test (*p* = 0.028) (Table 3). Again, we wanted to examine the impact on children who were not able to meet the state pre-K standard of balancing for 5 s at pre-test. The number of children who switched from balancing less than 5 s (did not meet the standard) to greater than 5 s (met the standard), did not increase significantly in the comparison group. However, the number of children did increase significantly in the intervention group (*p* = 0.007) (Table 3).

Boxing (crossing the midline). With regards to the entire COMPARISON study, the intervention group had a greater percentage of those who were competent in crossing the midline post-intervention than the comparison group (69%, 31%, respectively). With regards to children who were unable to complete the skill at pretest, crossing the midline improved significantly for both the intervention (pre-test 70 children to post-test 21; *p* < 0.001) and comparison groups (pre-test 105 children to post-test 53; *p* < 0.001).

Hops (number in 15 s). The number of hops on one foot in 15 s increased significantly from a mean of 24.25 (SD = 11.65) hops pre intervention to 28.49 (SD = 10.53) hops post-test in the intervention group (t = −3.545, *df* = 112, *p* = 0.001). The mean number of hops did not significantly increase in the comparison group (*p* = 0.064).

### 3.2. SCALE-UP Results

Table 4 shows descriptive results of the sample from the SCALE-UP study. The mean age of the children was 4.16 years, and 51.1% were female. The majority was Hispanic/Latino (54.1%). The mean BMI percentile was the 66th percentile and 34.7% were obese or overweight, which is slightly higher than Nevada’s obese/overweight preschool population (32%) [41]. In the SCALE-UP results, we consider first pre-intervention differences only. Then we consider post-intervention differences. In our post hoc analyses we use Wald tests to compare 12-skill, boxing, balance and hops scores pre- and post-intervention both with and without controlling for age. Lastly, Wald tests were run between boys and girls comparing the difference in the four scores from pre- to post-intervention. A Bonferroni adjustment was applied to maintain a familywise Type I error rate of α_fw_ = 0.05. With this adjustment, the slope (hierarchical linear model) or chi-square (Wald test) was viewed as statistically significant only if the *p*-value was less than α_adj_ = 0.0125.

#### 3.2.1. Pre-Intervention Differences

Pre-intervention scores were investigated whereby children were considered nested within one of the 28 ECE sites. For each task (sum 12-skills score, boxing score, number of hops and balance time), the unconditional model (intercept-only) was run to determine the proportion of variance explained by site alone. Then demographics (sex, age, ethnicity, and BMI z-scores) were added to the model. 

For the sum 12-skills score, the ICC showed that 9.56% (*p* = 0.010) of the total variation was accounted for by site. When the demographics were added to the model, age was significant (*b* = 1.269, *se* = 0.201, *p* < 0.001), ethnicity was significant (*b* = −1.442, *se* = 0.309, *p* < 0.001), and the ICC decreased to 4.94% (*p* = 0.027). These slopes imply that for each year increase in age, children complete more than one additional skill on average, and Hispanic children on average complete nearly one and a half fewer skills. 

For the boxing score, the ICC showed that 1.96% (*p* = 0.246) of the total variation was accounted for by the site. When demographics were added to the model, age was significant (*b* = 0.067, *se* = 0.015, *p* < 0.001), sex was significant (*b* = 0.065, *se* = 0.017, *p* < 0.001), and the ICC decreased to 1.05% (*p* = 0.367). These slopes confirm that older children and boys perform significantly higher on boxing prior to the intervention.

For the number of hops, the ICC showed that 6.06% (*p* = 0.014) of the total variation was accounted for by site. When the demographics were added to the model, sex was not significant (*b* = 1.370, *se* = 0.586, *p* = 0.020), age was significant (*b* = 4.933, *se* = 0.690, *p* < 0.001), BMI was significant (*b* = −0.692, *se* = 0.260, *p* = 0.008), and the ICC decreased to 6% (*p* = 0.05). Overall, these slopes show that boys on average complete more hops, and children with higher BMI complete fewer hops on average. Interestingly, for each year older, children on average complete approximately five more hops.

For the timed balance, the ICC showed that 4.52% (*p* = 0.085) of the total variation was accounted for by site. When demographics were added to the model, age was significant (*b* = 1.974, *se* = 0.342, *p* < 0.001), ethnicity was significant (*b* = −1.113, *se* = 0.314, *p* < 0.001), BMI was not significant (*b* = −0.14, *se* = 0.061, *p* = 0.022) and the ICC decreased to 0.84% (*p* = 0.140). These slopes show that on average for each year older, children balance for nearly two more seconds. Conversely, Hispanic children and children with a higher BMI present on average balance for fewer seconds.

#### 3.2.2. Post-Intervention Differences

Post-intervention scores were investigated whereby children were considered nested within one of the 28 ECE sites. For each task (sum 12-skills score, boxing score, number of hops and balance time), the unconditional model (intercept-only) was run to determine the proportion of variance explained by site alone. Then demographics (sex, age, ethnicity and BMI z-score) and the pre-intervention score were added to the model.

For the sum 12-skills score, the ICC showed 16.52% (*p* = 0.007) of the total variation was accounted for by site. When the demographics and the pre-12-skills score were added to the model, the pre-12-skills sum score was significant (*b* = 0.517, *se* = 0.026, *p* < 0.001), age was significant (*b* = 0.608, *se* = 0.117, *p* < 0.001), ethnicity was significant (*b* = −0.758, *se* = 0.161, *p* < 0.001) and the ICC decreased to 5.38% (*p* = 0.013). These slopes show that on average a child gained half of a skill by completing the intervention. In addition, older children continued to score higher on average, whereas Hispanic children continued to score lower on average.

For the post-intervention boxing score, the ICC showed that 3.74% (*p* = 0.209) of the total variation was accounted for by site. When demographics and the pre-boxing score were added to the model, the pre-boxing score was significant (*b* = 0.425, *se* = 0.037, *p* < 0.001), age was significant (*b* = 0.155, *se* = 0.027, *p* < 0.001), sex was significant (*b* = 0.053, *se* = 0.020, *p* = 0.010), ethnicity was not significant (*b* = −0.051, *se* = 0.022, *p* = 0.021), and the ICC decreased to 1.10% (*p* = 0.242). These slopes show that on average children increased their boxing score by nearly half a point. However, on average, older children, boys, and non-Hispanic children continued to score significantly higher in boxing.

For the number of hops, the ICC showed that 5.73% (*p* = 0.032) of the total variation was accounted for by site. When demographics and the pre-hops score were added to the model, the pre-hop score was significant (*b* = 0.454, *se* = 0.021, *p* < 0.001), age was significant (*b* = 2.208, *se* = 0.618, *p* < 0.001), sex was significant (*b* = 1.528, *se* = 0.317, *p* < 0.001), and the ICC decreased to 1.55% (*p* = 0.101). These slopes confirm that on average children score nearly half a hop more after the intervention. However, older children and boys continue to complete significantly more hops than younger children and girls, respectively.

For the timed balance, the ICC showed that 2.21% of the total variation was accounted for by site. When demographics and the pre-balance time were added to the model, the pre-balance time was significant (*b* = 0.473, *se* = 0.036, *p* < 0.001), age was significant (*b* = 2.084, *se* = 0.487, *p* < 0.001), and the ICC decreased to 2.08% (*p* =.131). These slopes show that on average children significantly increased their balance time with the intervention; however, older children on average continued to balance longer than younger children.

#### 3.2.3. Post hoc Wald Test Comparisons

Finally, Wald tests were run by simply comparing the means of the pre-intervention and post-intervention scores for each task. Table 5 summarizes the results. On average, scores for all four tasks significantly increased after the intervention: sum 12-skills score ($\chi^{2}$ = 1520.39, *df* = 1, *p* < 0.001), boxing ($\chi^{2}$ = 611.95, *df* = 1, *p* < 0.001), number of hops ($\chi^{2}$ = 323.58, *df* = 1, *p* < 0.001) and time balancing ($\chi^{2}$ = 129.23, *df* = 1, *p* < 0.001).

However, since age was consistently significant for all tasks, Wald tests were run with age as a covariate. Table 6 shows the results when controlling for age. When controlling for age pre-intervention and post-intervention scores were significantly different for the sum 12-skills score ($\chi^{2}$ = 31.02, *df* = 1, *p* < 0.001), boxing ($\chi^{2}$ = 11.64, *df* = 1, *p* = 0.001) and the number of hops ($\chi^{2}$ = 12.21, *df* = 1, *p* = 0.001).

Lastly, Wald tests were run between boys and girls comparing the difference in scores from pre- to post-intervention. No significant differences were detected for any task. Consequently, the intervention does not seem to benefit boys or girls more.

## 4. Discussion

It was hypothesized that intervention children would show greater improvements in movement skills than the effects of development alone (COMPARISON study) and that preschoolers would significantly improve their movement skills (SCALE-UP study). As noted above, *All 4 Kids*^©^ significantly improved all aspects measured in participating children. These results were not surprising as interventions tend to be effective [15,26,27]. However, a finding not typically explored, but much warranted, were the differential effects of biologic sex, ethnicity, BMI and age on outcomes of interest. Historically, young girls tend to struggle more with their movement competence than young boys [24]. These differences exist despite the fact that there are no anthropometric and physiological differences based upon biologic sex until puberty, [42] indicating something in the environment is posing as a constraint to development [23]. Thus, while a notable finding in the *All 4 Kids^©^* program, interventions should support the development for both boys and girls without differential effects.

*All 4 Kids^©^* utilizes dance as the primary activity because, during the preschool years, music and dance is acceptable for any gender and is enjoyable for children, their teachers and their families [43]. Dance focuses on overall fundamental skills (as opposed to primarily object manipulation) that helps children, especially girls, expand their motor repertoire early in life [44,45,46]. Children build confidence through skill instruction and repetition. Children engage in dances both in the classroom and in the home providing numerous opportunities for skill repetition to promote skill mastery.

Additionally, *All 4 Kids^©^* allows for inclusion regardless of skill level (differentiated learning). Program impact focuses on those children who could not reach the standard (e.g., balance for 5 s) prior to the intervention. While emphasis in the intervention design is to improve skills for children who are less proficient, all children are consistently engaged and building competency. Children practice the dances regularly in their entirety, while lessons focus primarily on individual dance move or fundamental skill proficiency.

Results of this study are unique and provide much-needed contribution to the intervention literature. To the authors’ knowledge, this study is the first of its kind to intervene upon multiple measures of both motor coordination and development in a population of children that were seemingly typically developed (without documented disability). These multiple systems include static balance (balance measure), dynamic balance and postural control (hops), movement competence (12-skills), and crossing the midline (motor coordination and control). Furthermore, the nature of the intervention (dance and music) were all created in a manner to also support fun and enjoyment within a movement setting. Historically, the motor skill intervention literature [26,27,30] tends to focus on one or two aspects of child development (e.g., only gross motor skill development or increasing physical activity behaviors) but fails to recognize the known antecedents of gross motor skill development and physical activity behaviors (e.g., static and dynamic balance, postural control and motor coordination). Thus, this study contributes to the literature in a meaningful and pragmatic way.

In addition to measuring multiple systems, this study occurred in an ecologically valid manner and featured one of the largest sample sizes explored within the intervention literature base. This robust sampling strategy enabled the authors to use high powered, complex statistical procedures that most previous studies are not adequately powered to attempt. As a result, *All 4 Kids*^©^ was scaled up to >25 schools and nearly 3000 children benefitted from participation during the study period. The authors were able to extract the extent to which *All 4 Kids*^©^, as a curriculum, influenced outcomes, as well as other influential factors, such as site effects and teacher. Typically, variance remains unexplained in lesser powered studies that do not include hierarchical modeling. However, *All 4 Kids*^©^ appears to be a powerful and effective curricular strategy towards fostering multiple aspects of motor development.

### Limitations

As novel and impactful as these data are, this study was not without limitations. Providing a nested model enabled teasing out whether or not children would benefit from *All 4 Kids*^©^ and the extent to which the curriculum was robust enough to withstand any extant site effects (e.g., the teacher and/or the site [built environment]). However, this study cannot specifically explain how much of the site variance was attributed to the teacher, the built environment and/or other previously explored correlates (culture, family, etc.) within the social-ecological model. Additionally, in both studies (COMPARISON and SCALE-UP), while most participants were from low income households, including Head Start, the authors recognize that these results may not be generalizable to the total population.

The authors also recognize that although the PMA has demonstrated reliability [38], it would be beneficial to test the instrument with other populations both nationally and internationally. Finally, while site was accounted for assessors were not. There is the potential that differences in program assessors may play an influential role in the outcomes. There is a future need to repeat the study to address these issues and explore more details within the intervention itself to provide specific evidence-based recommendations for maximizing learning and for precision with its delivery. However, the consistency of findings over time suggest the strength of *All 4 Kids*^©^ in improving movement skills.

## 5. Conclusions

Directed movement resulted in improvements much greater than natural development between groups (intervention and comparison groups) and across time in a subsample of preschoolers (COMPARISON). Additionally, in the *All 4 Kids*^©^ large-scale intervention using directed repetition (SCALE-UP) improvements across time in movement skills (12-skills, balance time, hopping product and crossing the midline) were noted in those preschoolers enrolled. The *All 4 Kids*^©^ physical development intervention was initially created to measure the extent to which children within the state of Nevada met state-based standards, learning outcomes and expectations. This project is unique in this aspect as Nevada is one of the few states to recognize the importance of motor development and place guidelines surrounding children’s age-based expectations. The authors recommend that other states, as well as other US and international organizations, consider adopting similar specific policies.

In addition to being aligned with state standards, *All 4 Kids*^©^ was developed so teachers could deliver it in a manner that elicited meaningful change for preschoolers. These findings suggest that other entities could consider requiring similar motor development curricular changes to ECE programs that receive public funds (e.g., Head Start, public schools). If such entities place an intervention such as *All 4 Kids*^©^ within preschool curricula, remediation of the secular motor skill decline (and developmental skill delays) [23] present among today’s children [23] may be achieved.

## Figures and Tables

**Table 1:** Population Characteristics, COMPARISON Study, *All 4 Kids*^©^ Program (*n* = 379).

Demographic Variable	Intervention Group (*n* = 236) *n* (%) *	Comparison Group (*n* = 143) *n* (%) *	Total *n* (%) *
**Sex**			
Male	76 (44.7)	41 (40.2)	117 (43.0)
Female	94 (55.3)	61 (59.8)	155 (57.0)
**Race/Ethnicity**			
Black/African American	34 (21.7)	13 (14.1)	47 (19.0)
Hispanic	109 (69.9)	67 (72.8)	176 (71.0)
White	11 (7.1)	10 (10.9)	21 (8.4)
Other	2 (1.2)	2 (2.2)	4 (1.6)
**Age (by year)**			
4	105 (58.7)	69 (59.0)	174 (58.8)
5	74 (41.3)	48 (41.0)	122 (41.2)
**BMI Percentile Category**			
Underweight (<5th)	3 (2.5)	2 (3.2)	5 (2.8)
Healthy Weight (5th–<85th)	66 (55.9)	31 (49.2)	97 (53.6)
Overweight (85th–<95th)	19 (16.1)	14 (22.2)	33 (18.2)
Obese (≥95th)	30 (25.4)	16 (25.4)	46 (25.4)

* Missing cases omitted from analysis.

**Table 2:** 12-Skills, COMPARISON Study, *All 4 Kids^©^* Program.

Children Not Able to Perform Skill at Pre-Test				
Skill	Intervention Group		Comparison Group	
	Pre-Test *n* (%)	Post-Test *n* (%), *p*-Value **	Pre-Test *n* (%)	Post-Test *n* (%), *p*-Value **
1. Jump high	Sample size was too small due to ceiling effect for jumping high at pre-test.			
2. Hop on 1 foot	47 (39.8)	34 (72.3), < 0.001 *	32 (41.0)	20 (62.5), 0.004 *
3. Spin on 1 foot	81 (75.7)	53 (65.4), < 0.001 *	59 (78.6)	16 (27.1), 0.093
4. Balance on 1 foot	66 (60.0)	38 (57.6), < 0.001 *	39 (50.6)	21 (53.8), 0.045 *
5. Cross feet	55 (47.4)	47 (85.4), < 0.001 *	37 (49.3)	18 (48.6), 0.043 *
6. Step forward	83 (78.3)	57 (68.7), < 0.001 *	49 (75.4)	20 (40.8), 0.002 *
7. Step back	63 (57.8)	50 (79.4), < 0.001 *	48 (65.8)	25 (52.1), 0.018 *
8. Step to the side	69 (65.7)	43 (62.3), < 0.001*	40 (56.3)	18 (45.0), 0.596
9. Move fast	36 (37.5)	27 (75.0), 0.005*	31 (49.2)	18 (58.1), 0.078
10. Move slow	42 (42.9)	26 (61.7), 0.082	36 (56.3)	10 (27.8), 0.999
11. Move up/high	36 (35.0)	27 (75.0), 0.009*	27 (39.7)	18 (66.7), 0.043 *
12. Move down/low	41 (40.6)	25 (61.0), 0.010*	37 (54.4)	17 (45.9), 0.064

* *p* < 0.05 is significant, calculated from Wilcoxon signed ranks tests. ** *p* < 0.05 is significant, calculated from McNemar tests.

**Table 3:** Balance on 1 Foot, COMPARISON Study, *All 4 Kids^©^* Program (*n* = 379).

Skill	Intervention Group			Comparison Group		
	$\bar{x}\;\mathrm{Secs}\;(\mathrm{SD})$	Median	*p*-Value *	$\bar{x}\;\mathrm{Secs}\;(\mathrm{SD})$	Median	*p*-Value *
Balance pre	5.32 (7.31)	2.59		4.92 (5.53)	2.9	
Balance post	6.42 (7.60)	3.2	0.028 *	4.67 (4.89)	2.73	0.485
**Children not Able To Balance for 5 s at Pre-Test**						
**Skillv**	**Pre-Test n (%)**	Post-Test n (%), *p*-Value **		**Pre-Test n (%)**	Post-Test n (%), *p*-Value **	
Balance	90 (76.3)	26 (28.9), 0.007 *		57 (77.0)	18 (31.6), 0.078	

* *p* < 0.05 is significant, calculated from Wilcoxon signed ranks tests. ** *p* < 0.05 is significant, calculated from McNemar tests.

**Table 4:** Population Characteristics, SCALE-UP Study, *All 4 Kids^©^* (*n* = 2817).

Demographic Variable	*n* (%) *
Sex	
Male	1158 (48.9)
Female	1209 (51.1)
Race/Ethnicity	
Black/African American	611 (25.4)
Hispanic	1301 (54.1)
White	304 (12.6)
Other	191 (7.9)
Age (by year)	
3	303 (14.4)
4	1158 (55.1)
5	631 (30.0)
6	10 (0.5)
BMI percentile	
Underweight (<5th)	59 (3.2)
Healthy Weight (5th–<85th)	1133 (62.1)
Overweight (85th–<95th)	310 (17.0)
Obese (≥95th)	322 (17.7)

* Missing cases omitted from analysis.

**Table 5:** Wald test for SCALE-UP *All 4 Kids^©^.*

Skill	(*n*)	Pre-Intervention	Post-Intervention	Chi-Square	*p*-Value
12-Skill_sum score	2150	5.215	7.662	1520.393	<0.001
Boxing	2137	0.115	0.377	611.954	<0.001
Hops	2122	20.107	25.26	323.576	<0.001
Balance	2128	5.56	7.428	129.227	<0.001

*Note.* Wald test comparing 12-skill, boxing, balance and hops scores pre- and post-intervention for the SCALE-UP phase.

**Table 6:** Wald test for SCALE-UP controlling for age, *All 4 Kids^©^.*

Skill	Pre-Intervention	Post-Intervention	Chi-Square	*p*-Value
12-Skill_sum score	−0.267	2.245	31.021	<0.001
Boxing	0.641	0.119	11.642	0.0006
Hops	−3.384	4.314	12.213	0.0005
Balance	−3.079	−4.921	2.262	0.1326

*Note.* Wald test comparing 12-skill, boxing, balance and hops scores pre- and post-intervention while controlling for age for the SCALE-UP phase.
